# AGE-FRIENDLY PUBLIC HEALTH SYSTEMS IN ACTION

**DOI:** 10.1093/geroni/igad104.0336

**Published:** 2023-12-21

**Authors:** Karon Phillips, Megan Wolfe

**Affiliations:** Trust for America’s Health, Washington, District of Columbia, United States; Trust for America’s Health, Washington, District of Columbia, United States

## Abstract

Trust for America’s Health (TFAH) leads the national Age-Friendly Public Health Systems movement, the goal of which is to elevate healthy aging as a core function of state and local departments of health. The nation’s public health system has contributed to the increased longevity of the population and there are numerous opportunities for public health to advance healthy aging in collaboration with the health care system, the aging services sector and others. TFAH has worked directly with state and local health departments to test the Age-Friendly Public Health Systems framework and seen first-hand the policy and systems changes that are possible through collective impact. During this symposium, participants will learn about the changes that are occurring in Mississippi and Washington state, where Age-Friendly Public Health practices are being adopted and implemented in collaboration with key partners, including expanding data collection on older adult health, ensuring older adults and caregivers are considered in emergency planning, and working directly with older adults in Tribal communities.

